# Integrated Network Pharmacology and Proteomics Reveal That Erxian Decoction Counteracts Postmenopausal Osteoporosis via GSTA1-Mediated Oxidative Stress Suppression

**DOI:** 10.3390/ph19050708

**Published:** 2026-04-30

**Authors:** Jingdi Li, Jiapo Zuo, Yaoting Zou, Yaoqiang Weng, Kaiyang Lin, Yihang Zou, Xuleqin Ye, Dezun Ma, Hui Yan

**Affiliations:** 1 Academy of Integrative Medicine, Fujian University of Traditional Chinese Medicine, No. 1, Qiu Yang Road, Shangjie, Minhou District, Fuzhou 350001, China; 13526972714@163.com (J.L.); 15073259172@163.com (J.Z.); 18759266287@163.com (Y.Z.); 18959175386@163.com (Y.W.); canlnk@163.com (K.L.); 13276972726@163.com (Y.Z.); 15882284076@163.com (X.Y.); 2 College of Integrative Medicine, Fujian University of Traditional Chinese Medicine, Fuzhou 350001, China; 3 Fujian Key Laboratory of Integrative Medicine on Geriatrics, Fujian University of Traditional Chinese Medicine, Fuzhou 350001, China

**Keywords:** Erxian Decoction, postmenopausal osteoporosis, oxidative stress, glutathione metabolism, GSTA1, proteomics

## Abstract

**Background**: Postmenopausal osteoporosis (PMOP) is a common metabolic bone disorder characterized by disrupted bone remodeling due to estrogen deficiency. Erxian Decoction (EXD), a traditional Chinese medicine formula, has shown clinical efficacy against PMOP, but its bioactive constituents and molecular mechanisms remain elusive. **Methods**: The therapeutic effects of EXD were evaluated in ovariectomized (OVX) mice using micro-CT and bone histomorphometry. Absorbed constituents of EXD were identified by UHPLC-Q analysis. Network pharmacology and quantitative proteomics were integrated to predict the key pathways and targets. The candidate target was validated by in vivo assays, including Western blot, glutathione (GSH) levels, glutathione S-transferases (GSTs) activity, malondialdehyde (MDA), and 4-hydroxynonenal (4-HNE) levels. Molecular docking was performed to assess the binding affinity between bioactive components and the target protein. **Results**: EXD treatment significantly ameliorated bone microarchitecture deterioration and restored bone remodeling balance in OVX mice. A total of 137 core absorbed constituents of EXD were identified. Integrated network pharmacology and proteomics analyses revealed that EXD primarily modulates the glutathione metabolism pathway to counteract oxidative stress. Glutathione S-transferase A1 (GSTA1) was pinpointed as a potential target. In vivo experiments confirmed that EXD upregulated GSTA1 expression, restored total GSTs activity, replenished GSH reserves, and reduced MDA and 4-HNE levels. Molecular docking demonstrated stable protein–ligand interactions between bioactive components of EXD and GSTA1. **Conclusions**: EXD alleviates PMOP by activating GSTA1-mediated glutathione metabolism and suppressing oxidative stress. These findings provide a mechanistic basis for the clinical application of EXD in treating PMOP.

## 1. Introduction

Postmenopausal Osteoporosis (PMOP) is a common chronic degenerative disease characterized by reduced bone mass and destruction of bone microarchitecture resulting from declining ovarian function [1,2]. With the accelerating aging of the global population, the prevalence of PMOP is increasing substantially [3]. The onset and progression of PMOP involve a multifactorial and multistage complex process—primarily driven by decreased estrogen levels, age-related bone remodeling imbalance, lipid metabolic disorders, and oxidative stress injury—which has been established as a critical pathological mechanism driving bone structural deterioration [4,5,6]. Pharmacotherapy remains the primary intervention for PMOP in clinical practice; however, its application is challenged by potential adverse effects and patient-specific factors [7]. Thus, there is an urgent need to develop effective therapeutic agents with fewer side effects.

Oxidative stress reflects an imbalance between the production of reactive oxygen species (ROS) and the antioxidant defense system, leading to oxidative damage to intracellular macromolecules including lipids, proteins, and DNA [8]. It plays a pivotal role in the pathogenesis of osteoporosis [9]. 4-Hydroxy-2-nonenal (4-HNE) and malondialdehyde (MDA) are terminal products of oxidative stress and serve as key molecular links between oxidative stress and disrupted bone metabolism [10,11,12,13]. Glutathione S-transferases (GSTs) act as key detoxifying enzymes of oxidative stress products by conjugating toxic electrophiles (e.g., 4-HNE and MDA) with glutathione (GSH) [14,15] thereby enhancing their water solubility and reducing cytotoxicity, ultimately suppressing oxidative stress in vivo. Therefore, inhibiting oxidative stress represents a critical strategy to impede the pathological progression of PMOP.

Traditional Chinese medicine formulas possess unique and significant value in the management of chronic diseases, owing to their multi-target, multi-pathway mechanisms, mild and gradual effects, and favorable overall tolerability, leading to their widespread clinical application [16]. Erxian Decoction (EXD), a classic TCM formula composed of six herbs—*Curculigo orchioides* (Xianmao), *Epimedium* (Xianlingpi), *Morinda officinalis* (Bajitian), *Anemarrhena asphodeloides* (Zhimu), *Phellodendron chinense* (Huangbo), and *Angelica sinensis* (Danggui)—has been extensively used in clinical practice due to its estrogen-like anti-osteoporotic effects [17]. Furthermore, experimental studies have demonstrated that EXD exhibits marked antioxidant properties [18,19,20]. However, further research is still required to substantiate the role of EXD in alleviating PMOP through the inhibition of oxidative stress and related pathological processes.

In this study, the primary constituents of EXD and its therapeutic efficacy against PMOP were identified and validated using liquid chromatography mass spectrometry combined with an ovariectomized animal model. Subsequently, network pharmacology analysis of the absorbed components and proteomic analysis elucidated the mechanisms by which EXD alleviates PMOP. The current findings provide robust experimental evidence supporting the therapeutic and preventive potential of EXD against PMOP.

## 2. Results

### 2.1. EXD Alleviated OVX-Induced Bone Microstructural Deterioration

**Figure 1:**
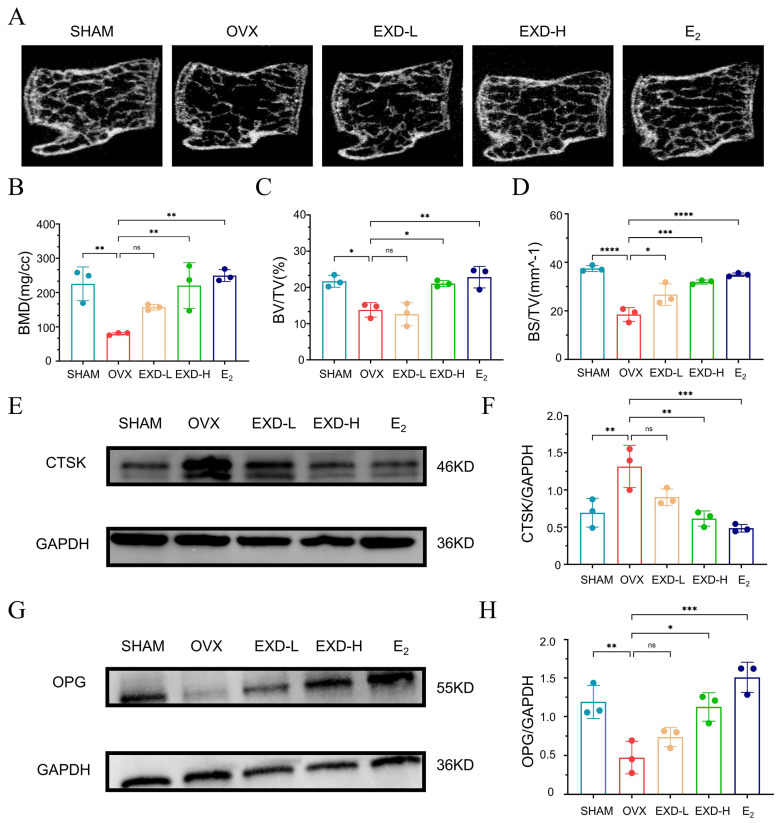
EXD alleviated OVX-induced microstructural deterioration of bone. (**A**) Representative micro-CT images of the lumbar vertebrae from the SHAM, OVX, EXD-L, EXD-H, and E_2_ groups. (**B**–**D**) Quantification of BMD (**B**), BV/TV (**C**) and BS/TV (**D**) in SHAM, OVX, EXD-L, EXD-H, and E_2_ groups (*n* = 3). (**E**–**H**) Western blot analysis of CTSK (**E**,**F**) and OPG (**G**,**H**) in bone tissues from SHAM, OVX, EXD-L, EXD-H, and E2 groups (*n* = 3). Relative expression of CTSK and OPG was normalized to GAPDH. All data are presented as mean ± SD from three independent experiments. ns, *p* > 0.05, * *p* < 0.05, ** *p* < 0.01, *** *p* < 0.001, **** *p* < 0.0001 versus OVX group.

To evaluate the therapeutic efficacy of EXD in postmenopausal osteoporosis (PMOP), an ovariectomized (OVX) mouse model was established using C57BL/6 mice. OVX mice were administered low or high doses of EXD (EXD-L and EXD-H groups, respectively) by gavage, with an estradiol (E2)-treated group serving as a positive control. Micro-CT analysis of the lumbar vertebrae revealed that OVX mice exhibited marked thinning, sparser, and structural disorganization of trabecular bone compared to the SHAM group. Notably, EXD-L treatment resulted in moderate improvement in bone microarchitecture, while EXD-H and E2 treatment led to more pronounced restoration (Figure 1A). Quantitative micro-CT parameters showed that OVX significantly reduced bone mineral density (BMD), bone volume fraction (BV/TV), and bone surface-to-tissue volume ratio (BS/TV). While EXD-H and E2 treatment significantly ameliorated all these parameters, EXD-L treatment only restored BS/TV without significantly improving BMD or BV/TV (Figure 1B–D). Furthermore, Western blot analysis of bone tissue revealed that EXD-H upregulated the expression of the osteoblastogenesis-associated marker osteoprotegerin (OPG) and downregulated the osteoclastogenesis-associated marker cathepsin K (CTSK) (Figure 1E–H). In summary, these findings show that EXD effectively alleviates PMOP.

### 2.2. Identification of the Principal Bioactive Constituents of EXD

**Figure 2:**
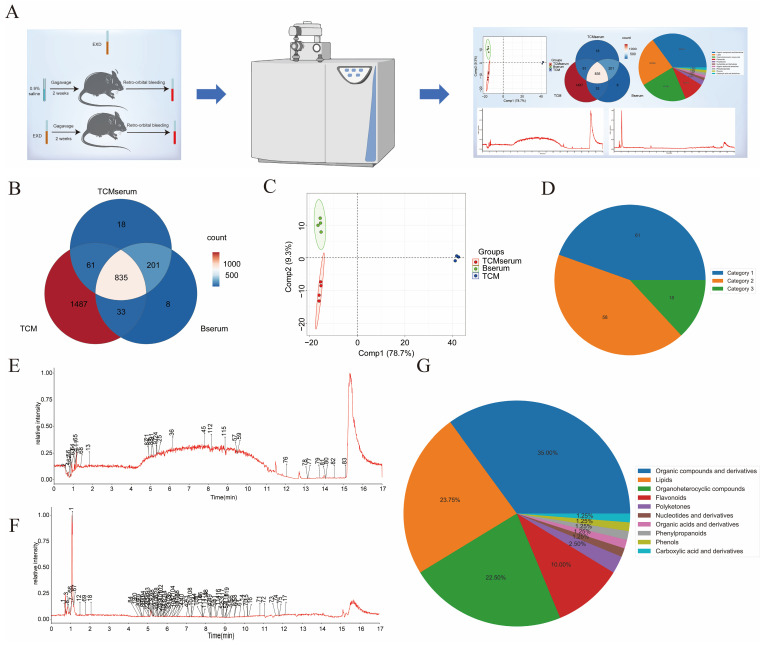
Identification of bioactive components absorbed from EXD. (**A**) Schematic diagram illustrating the workflow for identifying absorbed components of EXD. (**B**) Venn diagram showing the distribution of metabolites among the TCM, blank serum, and TCM-containing serum groups. (**C**) Principal component analysis (PCA) plots of the TCM, blank serum, and TCM-containing serum groups. (**D**) Hierarchical pie chart depicting the composition of blood-absorbed components from EXD. (**E**,**F**) Base peak chromatograms of blood-absorbed components from EXD obtained using UPLC-Q-TOF/MS in positive (**E**) and negative (**F**) ion modes. (**G**) Pie chart illustrating the classification of metabolites identified as blood-absorbed components of EXD.

To investigate the primary components responsible for the anti-PMOP effect of EXD, we performed UHPLC-Q analysis to identify the metabolite profiles of EXD samples (TCM), blank serum samples (Bserum), and EXD-containing serum samples (TCMserum) (Figure 2A). A total of 835 metabolites were detected across the three groups (Figure 2B). PCA revealed a clear separation between the TCMserum and TCM groups, indicating that EXD administration modulated the serum metabolite profile in mice (Figure 2C).

Given that many components of Er-Xian Decoction exert their effects only after being absorbed into the blood and subsequently distributed to target tissues and cells via the circulatory system, we compared the metabolites among the three groups and classified them into three categories. Category 1 metabolites were defined as those with peak areas greater than zero in both TCM and TCMserum but zero in Bserum. Category 2 metabolites were those present in all three groups, with a fold change (FC) of peak area between TCMserum and Bserum of ≥2 (FC TCMserum/Bserum ≥ 2). Category 3 metabolites were those detected exclusively in TCMserum, with peak areas of zero in both TCM and Bserum. A total of 137 principal bioactive constituents were identified (Figure 2D and Figure S1), comprising 61 Category 1, 58 Category 2, and 18 Category 3 metabolites (Figure 2E,F, and Table S1). Further classification of these metabolites revealed that organic compounds and their derivatives accounted for 35.00%, lipids for 23.75%, organic heterocyclic compounds for 22.50%, flavonoids for 10%, and polyketones for 2.50%, among other categories (Figure 2G).

### 2.3. Network Pharmacology Identifies Oxidative Stress as a Key Mechanism Underlying the Anti-PMOP Effect of EXD

**Figure 3:**
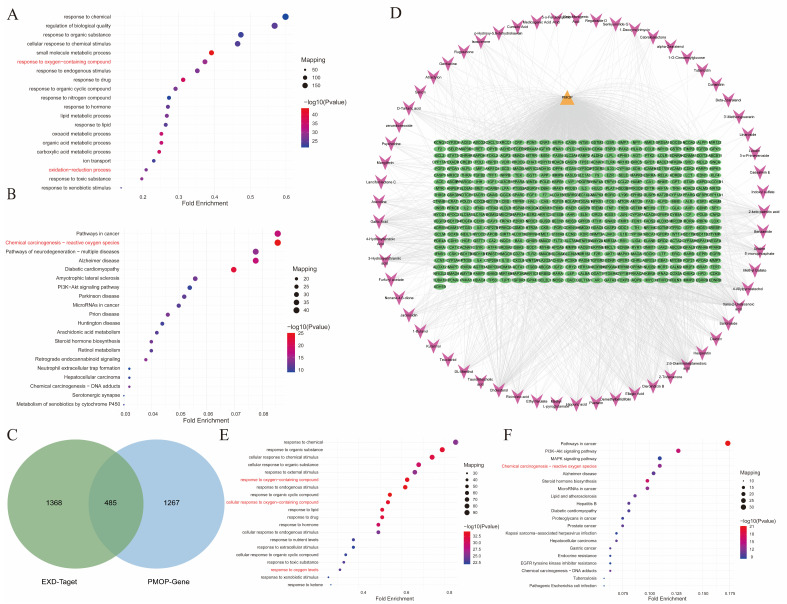
Network pharmacology-based identification of the key mechanisms underlying the anti-PMOP effects of EXD. (**A**) Top 20 biological processes enriched by the target genes of blood-absorbed components. (**B**) Top 20 KEGG pathways enriched by the target genes of blood-absorbed components. (**C**) Venn diagram showing the overlap between target genes of blood-absorbed components and PMOP-related target genes. (**D**) Component-target-disease network diagram illustrating the interactions among blood-absorbed components, their corresponding targets, and PMOP-associated targets. (**E**) Top 20 biological processes enriched by the overlapping targets between blood-absorbed components and PMOP. (**F**) Top 20 KEGG pathways enriched by the overlapping targets between blood-absorbed components and PMOP. Oxidative stress-related pathways are highlighted in red in both GO and KEGG analyses.

In order to further elucidate the potential mechanisms of EXD, the absorbed components were subjected to target gene prediction analysis using databases such as BATMAN-TCM and Binding DB. Based on the similarity scores between “absorbed component-target” and “known drug-target”, a total of 69 target genes corresponding to the absorbed components were identified using a cutoff score of ≥20 (Table S2). GO enrichment analysis of these target genes revealed significant enrichment in biological processes related to “response to oxygen-containing compound” and “oxidation-reduction process” (Figure 3A). Furthermore, KEGG pathway enrichment analysis demonstrated that the “Chemical carcinogenesis-reactive oxygen species” signaling pathway was prominently enriched (Figure 3B).

To further delineate the mechanisms underlying the therapeutic effects of EXD against PMOP, we intersected the PMOP-related target genes obtained from the Genecards database with the predicted target genes of the absorbed components. This analysis yielded 485 key genes in total (Figure 3C,D). GO enrichment analysis showed significant enrichment in terms associated with “response to oxygen-containing compound,” “cellular response to oxygen-containing compound,” and “response to oxygen levels” (Figure 3E). Consistently, KEGG pathway enrichment analysis revealed substantial enrichment of the “Chemical carcinogenesis-reactive oxygen species” signaling pathway (Figure 3F). These findings suggest that the active constituents of EXD may exert their therapeutic effects through an antioxidant stress mechanism.

### 2.4. Proteomic Analysis Reveals the Involvement of Oxidative Stress-Related Pathways in the Therapeutic Effect of EXD Against PMOP In Vivo

To further investigate the mechanistic basis of EXD treatment for PMOP in vivo, we performed proteomic profiling of femoral bone samples from the SHAM, OVX, and EXD-treated groups. Following data normalization, Pearson’s correlation coefficients among samples within each group were greater than 0.99, indicating high data reproducibility and appropriate sample selection (Figure S2). Differentially expressed proteins (DEPs) were identified based on the criteria of *p*-value < 0.05 and fold change (FC) > 1.3 or FC < 0.77.

**Figure 4:**
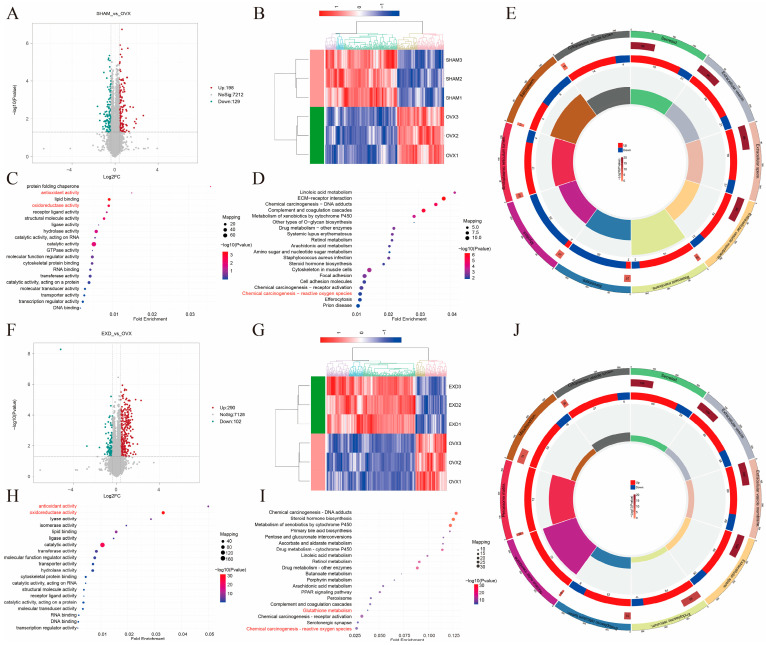
Proteomic analysis reveals oxidative stress-related pathways as key mechanisms underlying the therapeutic effects of EXD. (**A**,**B**) Volcano plot (**A**) and heatmap (**B**) of differentially expressed proteins (DEPs) between SHAM (*n* = 3) and OVX (*n* = 3) group. (**C**,**D**) Top 20 molecular functions (**C**) and top 20 KEGG pathways (**D**) enriched by upregulated DEPs in SHAM vs. OVX comparison. (**E**) Subcellular localization analysis of all DEPs enriched in SHAM vs. OVX comparison. (**F**,**G**) Volcano plot (**F**) and heatmap (**G**) of DEPs between EXD (*n* = 3) and OVX (*n* = 3) groups. (**H**,**I**) Top 20 molecular functions (**H**) and top 20 KEGG pathways (**I**) enriched by upregulated DEPs in EXD vs. OVX comparison. (**J**) Subcellular localization analysis of all DEPs enriched in the EXD vs. OVX comparison. Oxidative stress-related pathways are highlighted in red in both GO and KEGG analyses.

A total of 327 DEPs were identified in the SHAM vs. OVX comparison, comprising 198 upregulated and 129 downregulated proteins, as illustrated by the volcano plot and heatmap (Figure 4A,B). GO enrichment analysis of the upregulated proteins revealed significant enrichment in the molecular function term “oxidoreductase activity” (Figure 4C). KEGG pathway enrichment analysis indicated that the “Chemical carcinogenesis-reactive oxygen species” signaling pathway was significantly enriched (Figure 4D). GO and KEGG enrichment analyses of the downregulated proteins are presented in Figure S3A,B. Subcellular localization analysis showed that these DEPs were primarily enriched in secreted proteins, extracellular vesicle membranes, the basement membrane, extracellular vesicles, and the extracellular space (Figure 4E).

Proteomic analysis was also performed comparing the EXD and OVX groups (EXD vs. OVX), identifying a total of 392 DEPs, including 290 upregulated and 102 downregulated proteins. The expression changes are visually represented in the volcano plot and heatmap (Figure 4F,G). GO enrichment analysis of the upregulated proteins demonstrated significant enrichment in the molecular function terms “oxidoreductase activity” and “antioxidant activity” (Figure 4H). KEGG pathway analysis revealed significant enrichment of the “Glutathione metabolism” and “Chemical carcinogenesis-reactive oxygen species” signaling pathways (Figure 4I). Enrichment analyses of the downregulated proteins are shown in Figure S3C,D. Subcellular localization analysis indicated that these DEPs were predominantly localized to mitochondria and peroxisomes, closely associated with oxidative stress regulation (Figure 4J). Being consistent with the network pharmacology findings, the proteomic data confirm that the therapeutic effects of EXD are significantly associated with the enrichment of antioxidant-related pathways.

### 2.5. Central Role of Glutathione Metabolism in the Antioxidant Effect of EXD

**Figure 5:**
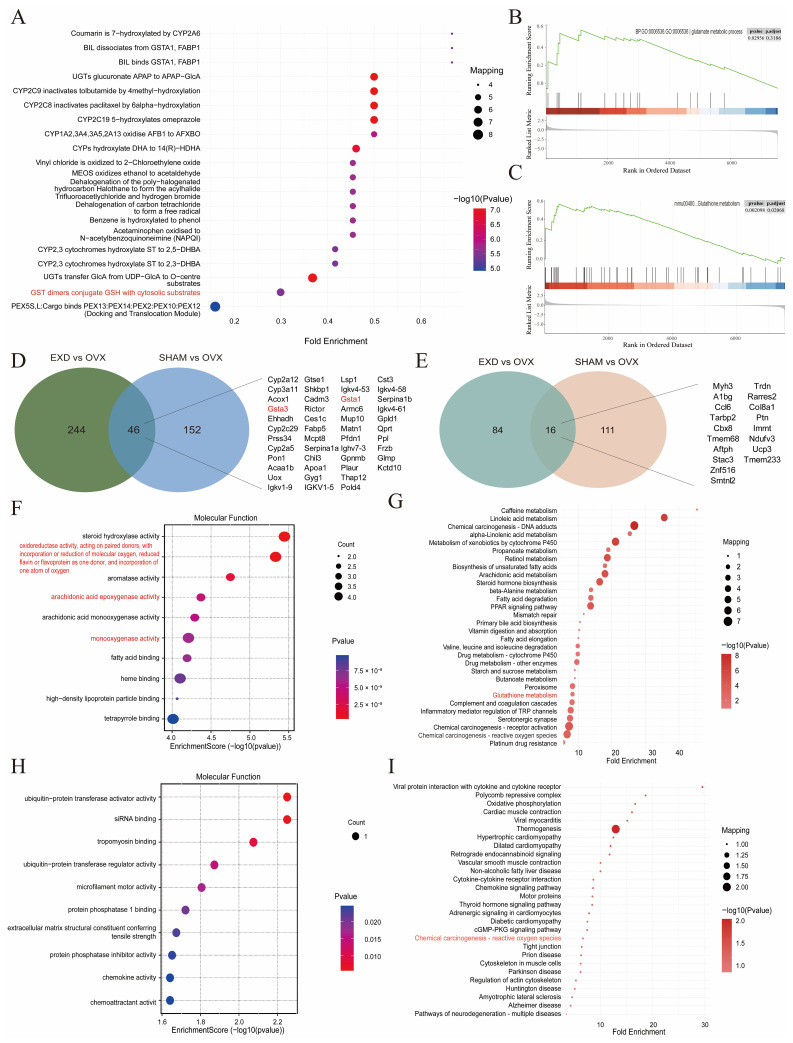
Glutathione metabolism plays a central role in the antioxidant mechanism of EXD. (**A**) Top 20 Reactome pathways enriched by DEPs in EXD vs. OVX comparison. (**B**,**C**) PSEA of the glutathione metabolism pathway in GO (**B**) and KEGG (**C**) analyses, showing significant upregulation of glutathione metabolism following EXD intervention compared with OVX group. (**D**,**E**) Venn diagrams showing proteins consistently upregulated (**D**) and consistently downregulated (**E**) in both SHAM vs. OVX and EXD vs. OVX comparisons. (**F**,**G**) Top 10 molecular functions (**F**) and top 30 KEGG pathways (**G**) enriched by proteins consistently upregulated in both SHAM vs. OVX and EXD vs. OVX comparisons. (**H**,**I**) Top 10 molecular functions (**H**) and top 30 KEGG pathways (**I**) enriched by proteins consistently downregulated in both SHAM vs. OVX and EXD vs. OVX comparisons. In Reactome, GO, and KEGG analyses, oxidative stress-related pathways are highlighted in red.

To elucidate the key molecular mechanisms underlying the antioxidant effect of EXD, we performed Reactome pathway enrichment analysis on the DEPs identified in the EXD vs. OVX comparison. This analysis provided more precise mechanistic evidence, revealing significant enrichment of the key reaction process “GST dimers conjugate GSH with cytosolic substrates” (Figure 5A). SPEA independently confirmed that this pathway was significantly upregulated following EXD intervention (Figure 5B,C). These mutually corroborating findings provide strong evidence supporting the critical role of glutathione metabolism in the antioxidant function of EXD.

For identifying the key targets through which EXD exerts its therapeutic effects, we performed an intersection analysis of DEPs that were either concurrently upregulated or downregulated in both the SHAM vs. OVX and EXD vs. OVX comparisons. This analysis yielded a total of 62 overlapping DEPs, comprising 46 upregulated and 16 downregulated proteins (Figure 5D,E). GO enrichment analysis of these overlapping DEPs revealed that the upregulated proteins were primarily enriched in terms related to “oxidoreductase activity,” “monooxygenase activity,” and “arachidonic acid epoxygenase activity” (Figure 5F), whereas the downregulated proteins were enriched in terms such as “ubiquitin-protein transferase activator activity,” “siRNA binding,” and “chemokine activity” (Figure 5G). KEGG pathway analysis demonstrated that the upregulated proteins were again significantly enriched in the “Glutathione metabolism” pathway, with GSTA1 and GSTA3—two key enzymes involved in glutathione regulation—being prominently enriched (Figure 5H). The downregulated proteins were significantly enriched in the “Chemical carcinogenesis−reactive oxygen species” pathway (Figure 5I). Based on these findings, we propose that GSTA1 and GSTA3 may serve as key targets through which EXD intervenes in glutathione metabolism.

### 2.6. GSTA1 Mediates the Antioxidant Effect of EXD by Restoring Glutathione Homeostasis in PMOP

**Figure 6:**
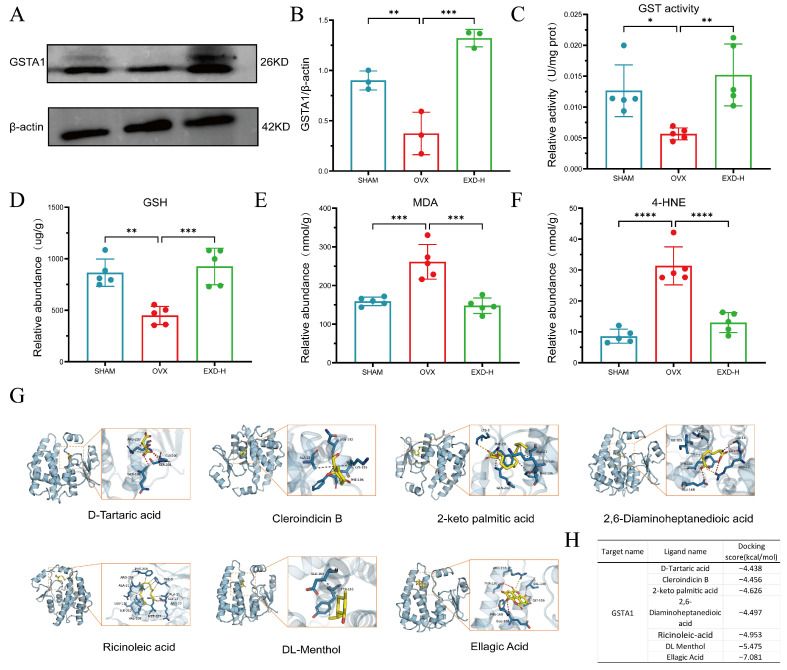
EXD alleviates oxidative stress and treats PMOP by targeting GSTA1. (**A**,**B**) Protein levels of GSTA1 in the SHAM, OVX, and EXD-H groups were detected by Western blotting. Relative expression of GSTA1 was normalized to β-actin (*n* = 3). (**C**) GST enzyme activity in the tibiae of the SHAM, OVX, and EXD-H groups (*n* = 5). (**D**–**F**) Levels of GSH (**D**), MDA (**E**), and 4-HNE (**F**) measured in tibial tissues (*n* = 5). (**G**,**H**) Molecular docking results of GSTA1 with six components predicted to target GSTA1 in EXD, namely ellagic acid, 2-ketopalmitic acid, DL-menthol, 2,6-diaminoheptanedioic acid, D-tartaric acid, cleroindicin B, and ricinoleic acid (**G**). The corresponding binding energies are summarized in the table (H). All data are presented as mean ± SD. * *p* < 0.05, ** *p* < 0.01, *** *p* < 0.001, **** *p* < 0.0001 versus OVX group.

GSTA1 and GSTA3 were identified as key enzymes through which EXD intervenes in glutathione metabolism. Given that GSTA1 is primarily responsible for detoxification functions [21], we hypothesized that GSTA1 serves as a critical enzyme through which EXD exerts its anti-PMOP effect by regulating glutathione metabolism. To test this hypothesis, we first examined GSTA1 protein expression levels in bone tissues by Western blotting. The results showed that, compared with the SHAM group, GSTA1 expression was significantly downregulated in the OVX group, whereas EXD-H intervention markedly reversed this reduction (Figure 6A,B). Consistently, total GSTs enzyme activity assays revealed that GST activity was significantly decreased in bone tissues of the OVX group, and this reduction was restored upon EXD-H administration (Figure 6C).

Considering that GSTA1 catalyzes the conjugation of reduced glutathione (GSH) with oxidative stress products such as 4-hydroxynonenal (4-HNE) and malondialdehyde (MDA) to facilitate their clearance, we further measured the levels of GSH, MDA, and 4-HNE in bone tissues. The results demonstrated that, compared with the SHAM group, the OVX group exhibited significantly decreased GSH content and increased MDA and 4-HNE levels. Notably, EXD intervention effectively reversed these alterations, restoring GSH levels and reducing MDA and 4-HNE contents (Figure 6D–F).

We further identified seven bioactive absorbed components from EXD that were predicted to target GSTA1 based on database screening: Ellagic Acid, 2-keto palmitic acid, DL-Menthol, 2,6-Diaminoheptanedioic acid, D-Tartaric acid, Cleroindicin B, and Ricinoleic acid. Molecular docking further validated that all seven compounds exhibited strong binding affinity to GSTA1, with the lowest binding energies ranging from −4.438 to −7.081 kcal/mol, indicating a high potential for stable protein–ligand interactions (Figure 6G). Among these, Ellagic Acid and DL-Menthol demonstrated particularly low binding energies of −7.081 and −5.475, respectively (Figure 6H), indicating these two compounds as promising active constituents within EXD responsible for modulating glutathione metabolism. In a word, these findings establish GSTA1 as a key target protein through which EXD alleviates PMOP via antioxidant stress regulation.

## 3. Discussion

PMOP has emerged as a serious and growing global public health challenge, imposing a substantial healthcare burden on both patients and society. Considering the limitations of common therapies, traditional Chinese medicine (TCM) formulas offer unique advantages in the prevention and treatment of PMOP by virtue of their multi-target and multi-pathway regulatory characteristics. EXD, a classic and representative formula widely used in clinical practice for PMOP, has demonstrated well-defined therapeutic efficacy; however, its underlying mechanisms warrant further in-depth investigation. In this study, through in vivo efficacy evaluation and compositional analysis of the formula, we systematically investigated the therapeutic effects of EXD on PMOP and identified 137 core absorbed metabolites. Network pharmacology analysis further suggested that targeting oxidative stress represents a key mechanism underlying the anti-PMOP effects of EXD. Subsequent proteomics and experimental validation revealed that EXD significantly modulates glutathione metabolism and effectively inhibits oxidative stress by targeting glutathione S-transferase A1 (GSTA1), thereby exerting its therapeutic effects. To sum up, these findings provide further scientific evidence to support the clinical application of Erxian Decoction.

Numerous folk herbal remedies, as natural products, have been shown to be effective in the treatment of PMOP by promoting bone formation and inhibiting bone resorption through mechanisms such as pro-anabolic and anti-catabolic effects [22]. Erxian Decoction (EXD) is a classic formula composed of six medicinal herbs, namely *Curculigo orchioides* (Xianmao), *Epimedium* (Xianlingpi), *Morinda officinalis* (Bajitian), *Anemarrhena asphodeloides* (Zhimu), *Phellodendron chinense* (Huangbo), and *Angelica sinensis* (Danggui). In clinical practice, the application of EXD has primarily focused on the field of gynecological endocrinology, where it serves as a principal prescription for managing menopausal syndrome, including PMOP [23,24,25]. In the present study, using an ovariectomized PMOP animal model, we found that EXD treatment effectively ameliorated PMOP, as evidenced by improvements in both imaging findings and bone morphometric parameters. Furthermore, EXD intervention effectively restored the balance of bone metabolism by promoting bone formation and inhibiting bone resorption, which is consistent with previous reports [25].

The integrated application of network pharmacology and proteomics has been widely employed to elucidate the mechanisms of numerous traditional Chinese medicine formulas in the treatment of bone and joint diseases, such as Fufang Zhenshu Tiaozhi and Buqi Tongluo capsules. Following a similar analytical paradigm, our study adopted this integrated approach to investigate the therapeutic mechanism of EXD in the treatment of PMOP [26,27]. EXD is particularly recognized for its regulatory effects on the hypothalamic–pituitary–ovarian axis. Modern pharmacological studies have confirmed that EXD exerts phytoestrogen-like activities, thereby partially alleviating the bone metabolic imbalance resulting from postmenopausal estrogen deficiency. To further investigate the bioactive components of EXD, we identified the absorbed constituents in the blood, revealing a total of 137 active compounds. Bioinformatic analysis of the targets of these compounds showed significant enrichment in oxidative stress-related pathways, which aligns with previous reports highlighting the antioxidant and anti-inflammatory activities of EXD [28,29]. A category analysis of the active components revealed that organic compounds and their derivatives accounted for 35%, lipids accounted for 23.7%, organic heterocyclic compounds accounted for 22.5%, flavonoids accounted for 10%, and phenylpropanoids accounted for 2.5%. Notably, flavonoids are widely regarded as compounds with estrogen-like properties and have been reported to exert antioxidant effects [30,31]. For instance, the flavonoid-rich components in EXD, such as icariin and epimedin B from *Epimedium*, and mangiferin from *Anemarrhena asphodeloides*, have been shown to exert significant protective effects against oxidative stress injury through mechanisms including free radical scavenging and enhancement of antioxidant enzyme activity [32,33,34]. Moreover, the beneficial effects of these components on PMOP have been extensively documented.

Oxidative stress has been established as a significant pathogenic factor in the development of osteoporosis. The accumulation of ROS, diminished activity of antioxidant enzymes, and depletion of small-molecule antioxidants can profoundly disrupt bone homeostasis [4]. Nrf2 is a critical regulator of ROS. Notably, several active components identified in EXD, including ellagic acid and kaempferol, have been demonstrated to modulate the Nrf2 signaling pathway. These findings collectively suggest that the therapeutic effects of EXD are closely associated with the regulation of oxidative stress [19,35]. To investigate the specific biological processes and targets within oxidative stress pathways modulated by EXD, we performed proteomic analysis. Through KEGG pathway analysis, Reactome enrichment analysis, and SPEA of differentially expressed proteins following EXD intervention, glutathione metabolism emerged as a prominently implicated pathway in the antioxidant mechanism of EXD. Moreover, intersection analysis of differentially expressed proteins that were consistently upregulated or downregulated between the SHAM vs. OVX and EXD vs. OVX comparisons identified GSTA1 as a potential key regulatory target within glutathione metabolism. As one of the critical antioxidant defense systems in the body, glutathione metabolism plays an essential role in bone metabolism [36,37]. GSTs are key enzymes in this pathway, catalyzing the conjugation of reduced glutathione (GSH) with electrophilic substrates. They also possess glutathione peroxidase activity, enabling direct clearance of lipid peroxides, thereby exerting antioxidant effects. Our in vivo measurements confirmed that in the OVX group, GSTA1 expression and activity, as well as GSH levels, were significantly decreased, while oxidative stress markers such as MDA and 4-HNE were markedly elevated. Notably, EXD treatment significantly reversed these changes, indicating that EXD alleviates PMOP by modulating GSTA1 to regulate glutathione metabolism.

We further identified six bioactive components in EXD that were predicted to target GSTA1, namely ellagic acid, 2-ketopalmitic acid, ricinoleic acid, DL-menthol, 2,6-diaminoheptanedioic acid, D-tartaric acid, and cleroindicin B. Molecular docking analyses demonstrated stable binding interactions between these six compounds and GSTA1, with ellagic acid exhibiting the strongest binding affinity based on binding energy calculations. Ellagic acid has been extensively reported to inhibit bone loss in OVX animal models through its antioxidant effects [38,39]. Moreover, among the six identified molecules, ellagic acid is the only one that has been demonstrated to induce GST-Ya gene transcription and enhance its protein expression [40]. Therefore, we propose that ellagic acid may serve as a potential active component in EXD that targets GSTA1 to regulate glutathione metabolism in the treatment of PMOP.

Our study provides multifaceted evidence supporting the antioxidant mechanism of EXD; however, several limitations should be acknowledged. First, while our study establishes a strong correlation between GSTA1 expression and EXD efficacy, we have not formally proven causality. Knockdown of GSTA1 in vitro and in vivo to study its effect on PMOP is planned for our future investigations. Second, while we identified absorbed components in plasma, their local concentrations at the site of bone remain unknown.

## 4. Materials and Methods

### 4.1. Animals

Eight-week-old female C57BL/6 mice (*n* = 60) were purchased and housed at the Experimental Animal Platform for Technological Innovation and Translation, Fujian University of Traditional Chinese Medicine, under controlled conditions with humidity ranging from 40% to 70% and room temperature maintained at 22–25 °C. The mice were randomly assigned to the following groups: SHAM, ovariectomy (OVX), low-dose Erxian Decoction (EXD-L), high-dose Erxian Decoction (EXD-H), and estradiol (E2) groups. All mice in these groups underwent ovariectomy, whereas mice in SHAM group underwent a sham operation. Mice in the EXD-L and EXD-H groups were administered the drug at doses of 2.272 g·kg^−1^·day^−1^ and 4.545 g·kg^−1^·day^−1^, respectively, while mice in the E2 group received 10 mg·kg^−1^·day^−1^ of estradiol. SHAM and OVX mice were given an equivalent volume of normal saline for 8 weeks.

### 4.2. Animal Ethics

All mice were handled in accordance with the guidelines of the National Institutes of Health for the care and use of laboratory animals. All experimental procedures were approved and supervised by the Animal Care and Use Committee of Fujian University of Traditional Chinese Medicine (Approval No. 2025225).

### 4.3. Micro-CT Scanning

Lumbar vertebrae were fixed in 4% paraformaldehyde and subjected to micro-CT scanning. Scan images were generated and processed using a Quantum GX2 micro-CT imaging system (PerkinElmer, New York, NY, USA). Bone mineral density (BMD), bone volume fraction (BV/TV), and bone surface area to tissue volume ratio (BS/TV) were analyzed using Analyze 12.0 software.

### 4.4. Identification of the Composition of EXD In Vitro and In Vivo

#### 4.4.1. Preparation of EXD

*Curculigo orchioides* was purchased from Shandong Hongri Kangrentang Pharmaceutical Co., Ltd. (Jinan, China) (Batch No.: 0725003751). *Epimedium* (Batch No.: 0725003751), *Morinda officinalis* (Batch No.: 25003091), *Anemarrhena asphodeloides* (Batch No.: 24030831), *Phellodendron chinense* (Batch No.: 25020521), and *Angelica sinensis* (Batch No.: 25026871) were all purchased from Beijing Kangrentang Pharmaceutical Co., Ltd. (Beijing, China). The above medicinal materials were soaked in water for 30 min at a ratio of 3:3:3:2:2:3. After two decoctions, the final decoction was obtained. Subsequently, the extract was mixed, concentrated, dried, and sieved to obtain a powder suitable for storage.

#### 4.4.2. Collection of EXD Components in Blood

Eight-week-old female C57BL/6 mice were administered high-dose Erxian Decoction or an equivalent volume of normal saline for two weeks. Blood was collected from the orbital venous plexus. After standing for 2 h, the blood was centrifuged, and the supernatant was transferred to a centrifuge tube. An equal volume of extraction solution (methanol/acetonitrile, 1:1, *v*/*v*) was added, vortexed for 60 s, and subjected to low-temperature ultrasonic extraction for 30 min. The extraction was repeated twice. The sample was then placed at −20 °C for 1 h to precipitate proteins, and centrifuged at 12,000 rpm for 20 min at 4 °C. The supernatant was collected and vacuum-dried. The residue was reconstituted with 200 μL of 30% acetonitrile solution, vortexed, and centrifuged at 14,000 rpm for 15 min at 4 °C. An aliquot of 2 μL of the supernatant was used for analysis

#### 4.4.3. Mass Spectrometry and Chromatography Conditions

The extracted samples were analyzed using an Ultra-High Performance Liquid Chromatography–High Resolution Mass Spectrometry (UHPLC-HRMS, Thermo Scientific, Waltham, MA, USA). The analytical conditions were as follows: for UPLC, the column was a Waters HSS T3 column (100 × 2.1 mm, 1.8 μm); the column temperature was maintained at 40 °C; the flow rate was 0.3 mL/min; the injection volume was 2 μL; the solvent system consisted of phase A: Milli-Q ultrapure water containing 0.1% formic acid, and phase B: acetonitrile containing 0.1% formic acid; the gradient elution program was as follows: 0 min, A/B phase ratio 100:0, maintained until 1 min; at 12 min, the ratio changed to 5:95, maintained until 13 min; at 13.1 min, the ratio returned to 100:0, maintained until 17 min.

#### 4.4.4. Liquid Chromatography–Tandem Mass Spectrometry Analysis

High-resolution mass spectrometry data were acquired using a Q Exactive HF-X hybrid quadrupole-orbitrap high-resolution mass spectrometer (Thermo Fisher Scientific, Waltham, MA, USA) equipped with a heated electrospray ionization source in Full-ms-ddMS2 acquisition mode. The electrospray ionization source parameters were set as follows: sheath gas pressure at 40 arbitrary units; auxiliary gas pressure at 10 arbitrary units; spray voltage at +3000 V in positive ion mode and −2800 V in negative ion mode; ion source temperature at 350 °C; ion transfer tube temperature at 320 °C. The primary mass spectrometry scan range was set at a mass-to-charge ratio of 70–1050 Da, with a resolution of 70,000 for the full scan and 17,500 for the MS/MS scan.

### 4.5. Network Pharmacology Analysis

The absorbed components were imported into databases including TCMSP (https://www.tcmsp-e.com/tcmsp.php (accessed on 11 November 2025)), BATCM (http://bionet.ncpsb.org.cn/batman-tcm/index.php (accessed on 11 November 2025)), BindingDB (https://www.bindingdb.org/rwd/bind/index.jsp (accessed on 11 November 2025)), and Guide to Pharma to obtain target genes. Disease genes were retrieved from the GeneCards (https://www.genecards.org/ (accessed on 11 November 2025)) database using the keyword “postmenopausal osteoporosis”. These genes were further analyzed for Gene Ontology (GO) and Kyoto Encyclopedia of Genes and Genomes (KEGG) enrichment analyses.

### 4.6. Proteomics Analysis

#### 4.6.1. Sample Preparation

Bone tissue from the femur was lysed with lysis buffer to extract proteins. Protein concentration was determined using the BCA method, and protein quality was assessed by both concentration and quality, with visualization of protein bands via SDS-PAGE gel electrophoresis. Appropriate amounts of samples were subjected to protein denaturation, reduction, and alkylation. Subsequently, trypsin was added, and the samples were incubated at 37 °C for 2 h for enzymatic digestion. After digestion was terminated, the supernatant was collected for desalting using a C18 column, concentrated at 45 °C for later use, and then reconstituted for analysis.

#### 4.6.2. Mass Spectrometry Analysis

An appropriate amount of peptide from each sample was separated by chromatography using a Vanquish Neo UHPLC system (Thermo Scientific). Buffer A consisted of 0.1% formic acid in water, and buffer B consisted of 0.1% formic acid in 80% acetonitrile in water. The chromatographic column was equilibrated with 96% buffer A. Samples were loaded onto a trap column (PepMap Neo 5 µm C18 300 µm × 5 mm, Thermo Scientific) and subsequently separated on an analytical column (µPAC Neo High Throughput column, Thermo Scientific) using a gradient elution. The separated peptides were analyzed using an Orbitrap Astral mass spectrometer (Thermo Scientific) in data-independent acquisition (DIA) mode. The electrospray voltage was set to 1.9 kV in positive ion mode, with a precursor ion scan range of 380–980 *m*/*z*. The full scan resolution was 240,000, with an AGC target of 500% and a maximum injection time of 3 ms. For MS/MS scans, the resolution was 80,000, with an AGC target of 500%, a maximum injection time of 3 ms, and an RF-lens setting of 40%. MS2 activation type was HCD, with an isolation window of 2 Th, normalized collision energy of 25%, and a cycle time of 0.6 s.

#### 4.6.3. Data Analysis

DIA raw data files were analyzed using DIA-NN software (version 2.2). A proteomic reference database was used for the search. Peptide spectral libraries, generated through prediction and the MBR (match-between-runs) function, were used to extract quantitative protein information from the DIA raw data. Miss cleavages were set to 2, and trypsin was selected as the digestion enzyme. The final results were filtered at a 1% false discovery rate (FDR) at both the precursor ion and protein levels. The quantitative proteomic data obtained after filtering were subjected to advanced bioinformatics analyses. These methods included screening for differentially expressed proteins to identify changes in protein levels, COG (Clusters of Orthologous Groups) annotation for functional classification, subcellular localization analysis, and enrichment analyses including Gene Ontology (GO), Kyoto Encyclopedia of Genes and Genomes (KEGG), Protein Set Enrichment Analysis (PSEA), and Reactome pathway analysis. Differentially expressed proteins (DEPs) were identified based on the criteria of *p*-value < 0.05 and fold change (FC) > 1.3 or FC < 0.77.

### 4.7. Experimental Validation

#### 4.7.1. Measurement of Enzyme Activity

GST (glutathione S-transferase) activity was measured using a commercial kit according to the manufacturer’s protocol (GST Activity Assay Kit, KTB1630, Abbkine, Wuhan, China). One unit of GST activity was defined as the amount of enzyme from 1 mg of protein lysate from tibial tissue that catalyzed the conjugation of 1 µmol of 1-chloro-2,4-dinitrobenzene (CDNB) with GSH per minute at 37 °C.

#### 4.7.2. Measurement of Reduced Glutathione, Malondialdehyde, and 4-Hydroxynonenal

Levels of reduced glutathione, malondialdehyde, and 4-hydroxynonenal in the tibia were measured using commercial kits according to the manufacturer’s protocols (Reduced Glutathione Assay, KTB1600, Abbkine; Malondialdehyde Assay, A003-1-2, Nanjing Jiancheng, Nanjing, China; 4-Hydroxynonenal Assay, JRXW200086, Ruixin Biotech, Quanzhou, China).

#### 4.7.3. Western Blot Analysis

The femur or tibia was lysed using RIPA buffer (Epizyme, Shanghai, China) with a protease inhibitor (Beyotime, Shanghai, China) at a ratio of 100:1. Samples were shaken at 4 °C for 2 h and centrifuged at 14,000 rpm for 30 min, and the supernatant was collected. Protein quantification was performed using a bicinchoninic acid kit. Equal amounts of protein (20 μg) were separated by 10% SDS-PAGE and transferred onto PVDF membranes (Millipore, Burlington, MA, USA). The membranes were blocked in non-fat milk solution for 1 h at 25 °C and then incubated with primary antibodies overnight at 4 °C, followed by incubation with corresponding HRP-conjugated secondary antibodies for 1 h at room temperature. Immunoreactive proteins were visualized using an enhanced chemiluminescence immunoblotting detection kit (Abbkine, Wuhan, China). Band densities were quantified using Image Lab (version 6.1.0) or Image J software (version 1.53t). Antibodies used in immunoblotting included anti-GSTA1 (1:2000, 14475-1-P, Proteintech, Wuhan, China), anti-CTSK (1:2000, sc-48353, Santa Cruz, Dallas, TX, USA), anti-OPG (1:2000, 11534-1-AP, Proteintech, Wuhan, China), anti-GAPDH (1:50,000, 60004-1-lg, Proteintech, Wuhan, China), and anti-β-Actin (1:50,000, 66009-1-lg, Proteintech Wuhan, China).

### 4.8. Molecular Docking

Molecular docking was performed using AutoDock Vina software (version 1.2.3). Prior to docking, receptor proteins were processed using PyMol (version 2.5.5) to remove water molecules, ions, and small molecules. A docking box was then set to encompass the entire protein structure. Additionally, all processed small molecules and receptor proteins were converted into the PDBQT format required for AutoDock Vina using ADFRsuite. During docking, the global search exhaustiveness was set to 32, with other parameters kept at default settings. The docking conformation with the highest score was considered the binding conformation, and the docking results were visualized and analyzed using PyMol.

### 4.9. Statistical Analysis

Statistical analysis was performed using Prism 10. software (GraphPad Software Inc., La Jolla, CA, USA). Comparisons among multiple groups were conducted using one-way analysis of variance. Results are expressed as mean ± standard deviation, and a *p* value of < 0.05 was considered statistically significant.

## 5. Conclusions

The primary purpose of this study was to address the mechanistic gap in PMOP therapy by identifying the active components and molecular targets of EXD. In this study, we demonstrate that EXD exerts significant therapeutic effects against PMOP in ovariectomized mice. We further identified the absorbed bioactive components of EXD. Through integrated network pharmacology, proteomic analysis, and in vivo experimental validation, we identify glutathione metabolism as a core pathway mediating the therapeutic action of EXD against PMOP. In summary, these findings provide a solid scientific foundation for the clinical application of EXD.

## Data Availability

The original data presented in the study are openly available in Mendeley Data at DOI:10.17632/sjv3fwt626.1.

## Supplementary Materials

The following supporting information can be downloaded at: https://www.mdpi.com/article/10.3390/ph19050708/s1, Figure S1: Heat map of blood-absorbed metabolites of Erxian Decoction; Figure S2: Distribution and correlation analysis of protein quantitative values; Figure S3: Molecular functions and KEGG pathways enriched by downregulated DEPs; Table S1: The 137 metabolites identified as blood-absorbed components of Erxian Decoction; Table S2: The 69 blood-absorbed components identified with target genes.

## Abbreviations

The following abbreviations are used in this manuscript:

PMOP	postmenopausal osteoporosis
ROS	Directory of open access journals
4-HNE	4-Hydroxy-2-nonenal
MDA	malondialdehyde
GSH	glutathione
EXD	Erxian Decoction
EXD-L	low-dose Erxian Decoction
EXD-H	high-dose Erxian Decoction
E_2_	estradiol
OVX	ovariectomy
BMD	bone mineral density
BV/TV	bone volume fraction
BS/TV	bone surface area to tissue volume ratio
OPG	osteoprotegerin
CTSK	cathepsin K
TCM	EXD samples
Bserum	blank serum samples
TCMserum	EXD-containing serum samples
DEPs	Differentially expressed proteins
GSTA1	Glutathione S-transferase A1
GSTA3	Glutathione S-transferase A3
GSTs	glutathione S-transferases
GO	Gene Ontology
KEGG	Kyoto Encyclopedia of Genes and Genomes
PSEA	Protein Set Enrichment Analysis
