# Supplementary material for: Integrated Network Pharmacology and Proteomics Reveal That Erxian Decoction Counteracts Postmenopausal Osteoporosis via GSTA1-Mediated Oxidative Stress Suppression
Source: Pharmaceuticals (Basel). 2026 Apr 30;19(5):708. doi: 10.3390/ph19050708 (PMC13210001; doi:10.3390/ph19050708)
Supplement: Supplementary file 1 [file pharmaceuticals-19-00708-s001.zip › pharmaceuticals-4280938-supplementary.pdf]

SUPPLEMENTARY FIGURES

Supplementary figure 1

A

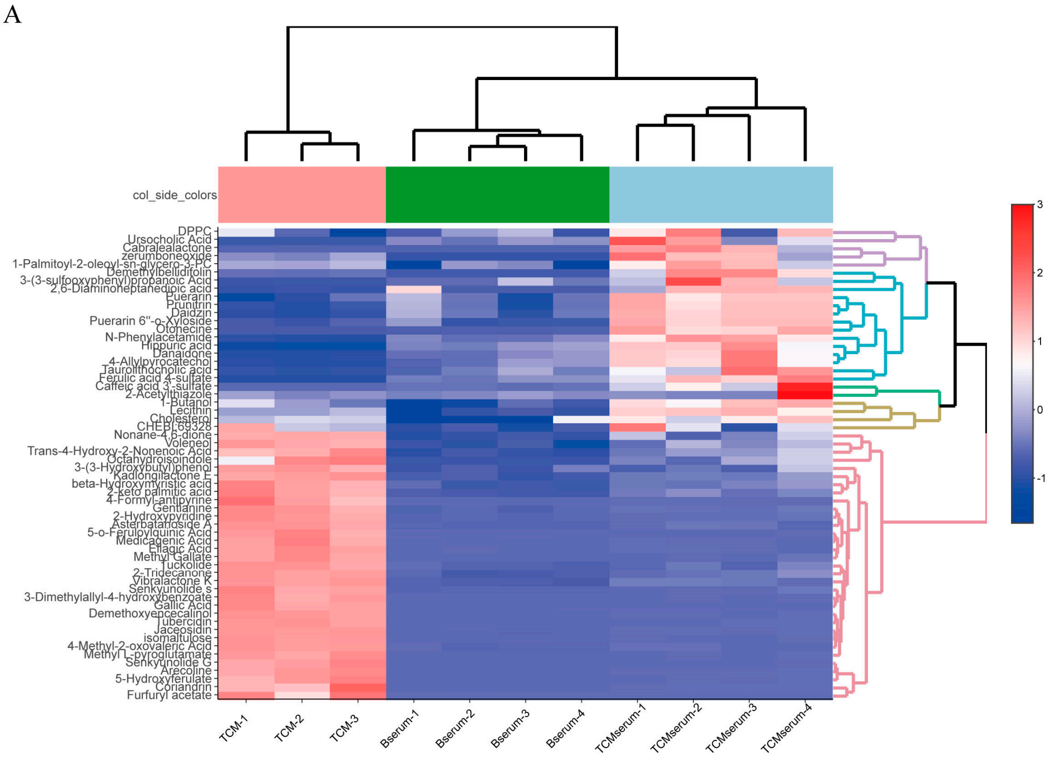

Figure S1. Heat map of blood-absorbed metabolites of Erxian Decoction. Heat map showing metabolites identified as blood-absorbed components of Erxian Decoction(A)

Supplementary figure 2

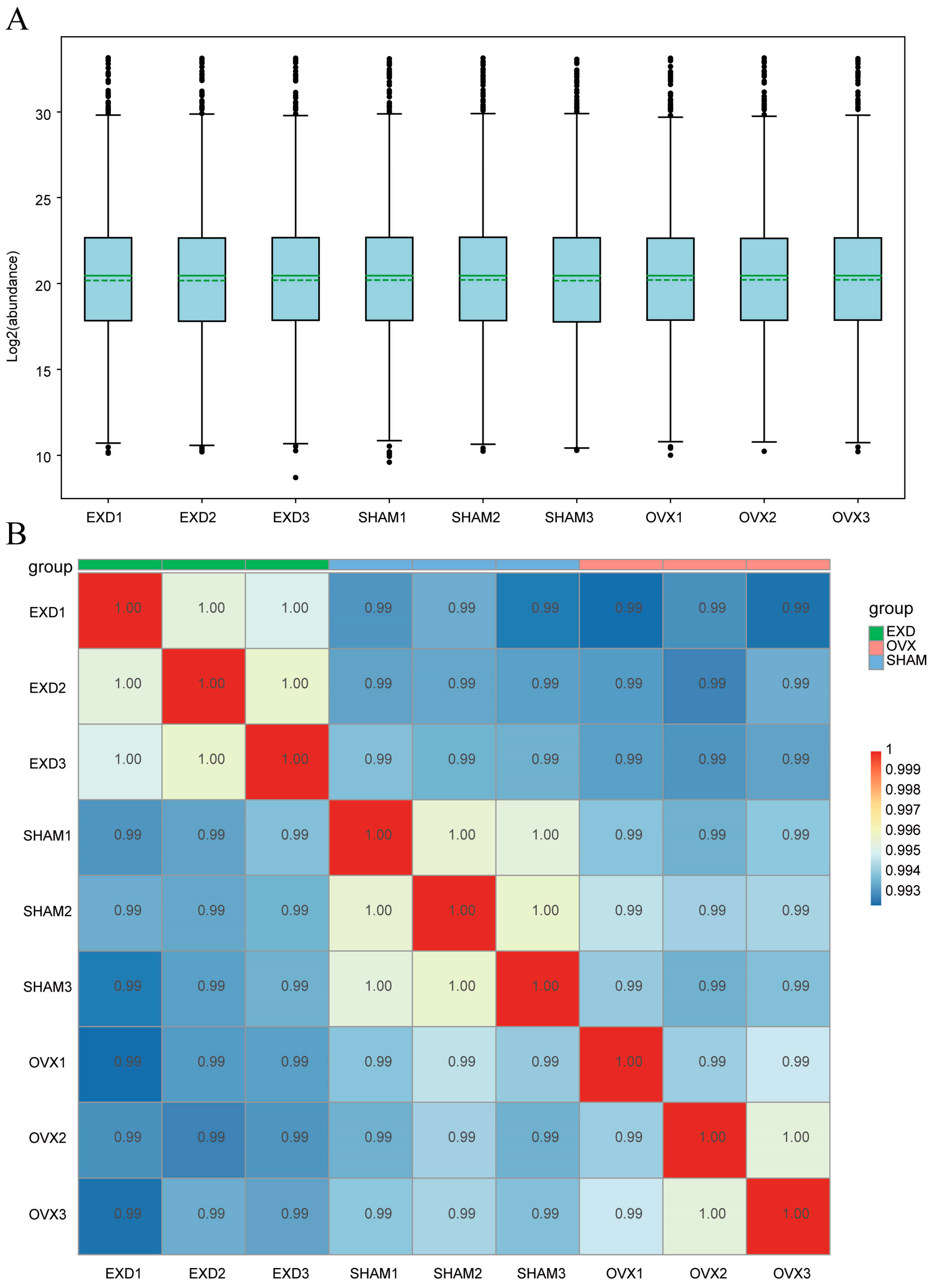

Figure S2. Distribution and correlation analysis of protein quantitative values. Box plot showing the distribution of normalized quantitative values of proteins in the SHAM, OVX, and EXD groups (A). Correlation analysis plot of protein data in the SHAM, OVX, and EXD groups (B).

Supplementary figure 3

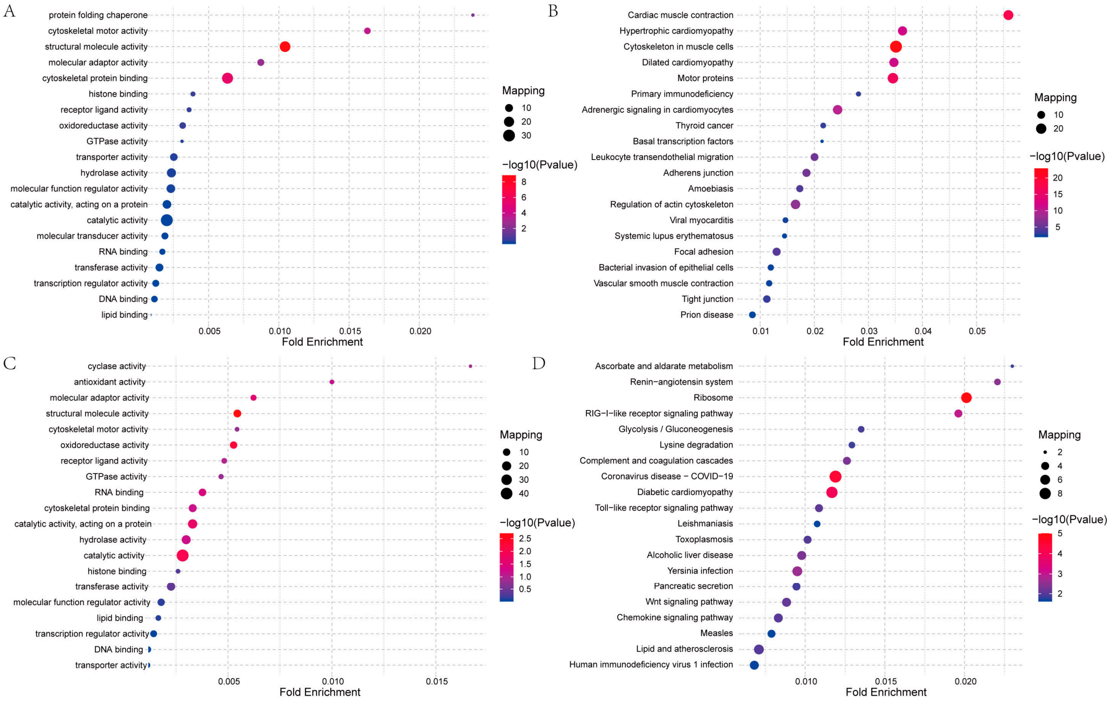

Figure S3. Molecular functions and KEGG pathways enriched by downregulated DEPs. (A-B) Top 20 molecular functions (A) and top 20 KEGG pathways (B) enriched by downregulated DEPs in SHAM vs. OVX comparison. (C-D) Top 20 molecular functions (C) and top 20 KEGG pathways (D) enriched by downregulated DEPs in EXD vs. OVX comparison.

Supplementary Table 1

The 137 metabolites identified as blood-absorbed components of Erxian Decoction.

Peak Number	Metabolite	Mode	m/z(Apex)	Formula	bloodmeta_class
1	D-Tartaric acid	neg	149.0093502	C4H6O6	Category 1
2	Ginnalin B	pos	380.0950013	C13H16O9	Category 1
3	Isosalvipuberulin	neg	379.0832914	C20H14O5	Category 1
4	7-Hydroxyflavanone-glucoside	pos	441.0913696	C21H22O8	Category 1
5	O-propanoyl-carnitine	pos	218.1383032	C10H19NO4	Category 1
6	Corey Lactone Diol	pos	190.1070276	C8H12O4	Category 1
7	Uridine 5'-monophosphate	neg	323.0293774	C9H13N2O9P	Category 1
8	Cumalic Acid	neg	139.0037897	C6H4O4	Category 1
9	Ethyl ferulate	pos	245.0764791	C12H14O4	Category 1
10	Periplanetin	neg	389.1098698	C13H16O7	Category 1
11	Ligustrosidic acid	pos	572.1972658	C25H30O14	Category 1
12	Benzoic acid + 2O, O-Hex	neg	315.0727231	C13H16O9	Category 1
13	Isopentyl b-D-glucoside	pos	250.1645203	C11H22O6	Category 1
14	LTB5	neg	315.1975175	C20H30O4	Category 1
15	Sclareol glycol	neg	313.2390392	C16H30O2	Category 1
16	11-Hydroxyundecanoic acid	neg	201.1500842	C11H22O3	Category 1
17	Cylindrol B	neg	351.1977306	C23H30O4	Category 1
18	1,2-O-Isopropylidene-alpha-D-fructopyranose	neg	279.1090464	C9H16O6	Category 1

19	Salicin	neg	345.11 99969	C13H18 O7	Category 1
20	Salidroside	neg	359.13 58316	C14H20 O7	Category 1
21	2-Isopropylmalic acid	pos	159.06 50179	C7H12O 5	Category 1
22	Caffeic acid 4-O-glucuronide	neg	337.05 71521	C15H16 O10	Category 1
23	1-O-Cinnamoylglucose	neg	415.12 57988	C15H18 O7	Category 1
24	Bruceantinol A	pos	592.23 8499	C29H36 O13	Category 1
25	3'-Methoxypuerarin	pos	447.12 81666	C22H22 O10	Category 1
26	Trp Glu Ile	neg	445.20 91634	C22H30 N4O6	Category 1
27	4-(2-Nitroethyl)phenyl primeveroside	neg	496.12 33797	C19H27 NO12	Category 1
28	Cleroindicin B	neg	203.09 29053	C8H14O 3	Category 1
29	Benzamide	neg	120.04 55438	C7H7NO	Category 1
30	3-Hydroxyanthranilic acid	neg	134.02 48923	C7H7NO 3	Category 1
31	Linaroside	neg	475.12 58169	C23H24 O11	Category 1
32	Lucidin 3-o-Primeveroside	neg	563.14 39632	C26H28 O14	Category 1
33	5-Chloro-6-methoxymellein	neg	277.00 27895	C11H11 ClO4	Category 1
34	Neochlorogenic Acid	neg	385.11 43091	C16H18 O9	Category 1
35	Regaloside D	neg	399.13 00011	C18H24 O10	Category 1
36	ORTH0-AMINOBENZOIC ACID	pos	138.05 47429	C7H7NO 2	Category 1
37	2,4,6-Trihydroxy-3-prenylacetophenone	neg	235.09 79654	C13H16 O4	Category 1
38	Mangiferin	neg	403.06 85457	C19H18 O11	Category 1
39	4-Hydroxybenzoic Acid	neg	275.05 39007	C7H6O3	Category 1
40	7,8-Dihydrokawain-5-ol	neg	353.12 53167	C14H16 O4	Category 1

41	Hesperetin	neg	301.07 22579	C16H14 O6	Category 1
42	Artonin M	neg	465.21 43202	C30H30 O7	Category 1
43	Tagitinin C	neg	329.14 03184	C19H24 O6	Category 1
44	p-Hydroxy-5,6-dehydrokawain	neg	275.09 27434	C14H12 O4	Category 1
45	Rugosinone	pos	354.09 68459	C19H15 NO6	Category 1
46	Hymatoxin A	neg	413.16 19179	C20H30 O7S	Category 1
47	PATULIN	neg	194.04 63127	C7H6O4	Category 1
48	Psychotrine	neg	445.24 60532	C28H36 N2O4	Category 1
49	DL-Menthol	neg	201.15 00377	C10H20 O	Category 1
50	Teuclatriol	neg	301.20 27222	C15H28 O3	Category 1
51	5-Hydroxy-7-methoxy-8-methylflavanone	neg	265.08 71382	C17H16 O4	Category 1
52	Monodictyxanthone	neg	251.03 53357	C15H10 O5	Category 1
53	(2E,4E,6Z)-Methyl deca-2,4,6-trienoate	neg	161.09 73963	C11H16 O2	Category 1
54	alpha-Zearalenol	neg	301.14 53316	C18H24 O5	Category 1
55	trans-2-Undecenoic acid	neg	165.12 87492	C11H20 O2	Category 1
56	Luteolin (Digitoflavone; Daphneflavonol; Flavopurpol; Luteolol)	neg	267.03 00881	C15H10 O6	Category 1
57	Isosclerone	pos	196.09 67184	C10H10 O3	Category 1
58	3-Hydroxychimaphilin	neg	201.05 61841	C12H10 O3	Category 1
59	1-Deoxynojirimycin	pos	204.08 55241	C6H13N O4	Category 1
60	Beta-Zearalanol	neg	303.16 08509	C18H26 O5	Category 1
61	2,9-Dihydroxy-7-methoxy-4-methylnaphtho[1,2-b]fura	neg	241.05 11042	C14H12 O5	Category 1
62	isomaltulose	pos	365.10 48522	C12H22 O11	Category 2

63	Arecoline	pos	156.10 16486	C8H13N O2	Category 2
64	5-Hydroxyferulate	pos	193.04 92919	C10H10 O5	Category 2
65	2,6-Diaminoheptanedioic acid	pos	232.12 87359	C7H14N 2O4	Category 2
66	Tubercidin	neg	265.09 31499	C11H14 N4O4	Category 2
67	Coriandrin	neg	321.06 25427	C13H10 O4	Category 2
68	2-Hydroxypyridine	pos	113.07 09574	C5H5NO	Category 2
69	Gallic Acid	neg	169.01 44807	C7H6O5	Category 2
70	2-Tridecanone	neg	243.19 69608	C13H26 O	Category 2
71	Medicagenic Acid	neg	501.32 31515	C30H46 O6	Category 2
72	2-keto palmitic acid	neg	269.21 25638	C16H30 O3	Category 2
73	Voleneol	neg	269.21 25608	C15H26 O2	Category 2
74	Kadlongilactone E	neg	507.27 40393	C31H40 O6	Category 2
75	beta-Hydroxymyristic acid	neg	243.19 69228	C14H28 O3	Category 2
76	Cabralealactone	pos	431.31 53373	C27H42 O4	Category 2
77	Cholesterol	pos	369.35 11021	C27H46 O	Category 2
78	Taurolithocholic acid	pos	448.29 01408	C26H45 NO5S	Category 2
79	1-Palmitoyl-2-oleoyl-sn-glycero-3-PC	pos	782.56 81029	C42H82 NO8P	Category 2
80	DPPC	pos	756.55 26692	C40H80 NO8P	Category 2
81	Lecithin	pos	758.56 85503	C42H80 NO8P	Category 2
82	Octahydroisoindole	pos	167.15 40036	C8H15N	Category 2
83	1-Butanol	pos	74.096 82152	C4H10O	Category 2
84	Methyl Gallate	neg	183.03 0293	C8H8O5	Category 2

85	2-Acetylthiazole	neg	126.00 20024	C5H5NO S	Category 2
86	Asterbatanoside A	neg	411.12 99471	C19H26 O11	Category 2
87	Furfuryl acetate	pos	141.05 44216	C7H8O3	Category 2
88	4-Formyl-antipyrine	pos	217.09 6828	C12H12 N2O2	Category 2
89	Gentianine	neg	215.08 30023	C10H9N O2	Category 2
90	5-o-Feruloylquinic Acid	neg	367.10 38853	C17H20 O9	Category 2
91	N-Phenylacetamide	pos	118.06 50373	C8H9NO	Category 2
92	Hippuric acid	neg	178.05 12514	C9H9NO 3	Category 2
93	Danaidone	neg	134.06 12769	C8H9NO	Category 2
94	Caffeic acid 3'-sulfate	neg	258.99 20407	C9H8O7 S	Category 2
95	Ferulic acid 4-sulfate	neg	273.00 76497	C10H10 O7S	Category 2
96	3-(3-sulfooxyphenyl)propanoic Acid	neg	245.01 28187	C9H10O 6S	Category 2
97	Puerarin	pos	417.11 76442	C21H20 O9	Category 2
98	Daidzin	neg	415.10 4935	C21H20 O9	Category 2
99	Puerarin 6''-o-Xyloside	neg	547.14 63117	C26H28 O13	Category 2
100	Prunitrin	neg	445.11 52047	C22H22 O10	Category 2
101	4-Methyl-2-oxovaleric Acid	neg	189.07 72214	C6H10O 3	Category 2
102	Tuckolide	neg	215.09 29628	C10H16 O5	Category 2
103	Trans-4-Hydroxy-2-Nonenoic Acid	neg	217.10 85759	C9H16O 3	Category 2
104	Otonecine	neg	184.09 82743	C9H15N O3	Category 2
105	Ellagic Acid	neg	300.99 97176	C14H6O 8	Category 2
106	Methyl L-pyroglutamate	neg	178.02 74596	C6H9NO 3	Category 2

107	Nonane-4,6-dione	neg	155.10 80433	C9H16O 2	Category 2
108	Demethylbellidifolin	neg	213.02 31299	C13H8O 6	Category 2
109	Senkyunolide s	neg	221.08 23137	C12H16 O5	Category 2
110	4-Allylpyrocatechol	neg	149.06 10024	C9H10O 2	Category 2
111	Demethoxyencecalinol	neg	249.11 35771	C13H16 O2	Category 2
112	CHEBI:69328	pos	295.15 36474	C16H22 O5	Category 2
113	Jaceosidin	neg	329.06 75279	C17H14 O7	Category 2
114	Ursocholic Acid	neg	407.28 15848	C24H40 O5	Category 2
115	zerumboneoxide	pos	427.25 98229	C15H22 O2	Category 2
116	3-(3-Hydroxybutyl)phenol	neg	331.19 23176	C10H14 O2	Category 2
117	3-Dimethylallyl-4-hydroxybenzoate	neg	205.08 72992	C12H13 O3-	Category 2
118	Senkyunolide G	neg	207.10 29226	C12H16 O3	Category 2
119	Vibralactone K	neg	209.11 86431	C12H18 O3	Category 2
120	Atractylon	pos	239.14 2622	C15H20 O	Category 3
121	Ricinoleic acid	pos	281.24 70611	C18H34 O3	Category 3
122	Indoxyl sulfate	neg	212.00 25936	C8H7NO 4S	Category 3
123	Mirificin	pos	549.15 9759	C26H28 O13	Category 3
124	Pseudoecgonine	pos	186.11 2114	C9H15N O3	Category 3
125	5-[(1S,2R,4aR)-5-(acetyloxymethyl)-1,2,4a-trimethyl-2,3,4,7,8,8a-hexahydronaphthalen-1-yl]-3-methylpentanoic acid	neg	423.27 6695	C22H36 O4	Category 3
126	S-Adenosyl-DL-Methionine	neg	457.15 16797	C15H22 N6O5S	Category 3
127	Danshenol C	neg	427.14 13431	C21H20 O4	Category 3

128	Digitoxigenin (Thevetigenin; Echujetin; Cerberigenin)	neg	405.2662671	C23H34O4	Category 3
129	Caesalmin E	pos	534.2734028	C26H36O9	Category 3
130	Pregn-5-Ene-3,17-Diol	neg	423.2766932	C21H34O2	Category 3
131	2-Desoxy-4-epi-pulchellin	pos	273.1479838	C15H22O3	Category 3
132	Kurarinol	pos	507.2621402	C26H32O7	Category 3
133	Negundonorin A	pos	469.2947105	C29H40O5	Category 3
134	Irgarol-descyclopropyl	pos	255.1375882	C8H15N5S	Category 3
135	3-hydroxy-12-oxochol-9(11)-en-24-oic acid	pos	389.268144	C24H36O4	Category 3
136	Syringolin C	pos	530.2962402	C25H41N5O6	Category 3
137	Lancifodilactone C	neg	525.2149983	C29H36O10	Category 3

Supplementary Table 2

The 69 blood-absorbed components identified with target genes.

Peak Number	Metabolite	Mode	m/z(Apex)	Formula	bloodmeta_class
1	D-Tartaric acid	neg	149.00935	C4H6O6	Category 1
7	Uridine 5'-monophosphate	neg	323.029377	C9H13N2O9P	Category 1
8	Cumalic Acid	neg	139.00379	C6H4O4	Category 1
9	Ethyl ferulate	pos	245.076479	C12H14O4	Category 1
17	Cylindrol B	neg	351.197731	C23H30O4	Category 1
19	Salicin	neg	345.119997	C13H18O7	Category 1
20	Salidroside	neg	359.135832	C14H20O7	Category 1
23	1-O-Cinnamoylglucose	neg	415.125799	C15H18O7	Category 1
25	3'-Methoxypuerarin	pos	447.128167	C22H22O10	Category 1
28	Cleroindicin B	neg	203.092905	C8H14O3	Category 1
29	Benzamide	neg	120.045544	C7H7NO	Category 1
30	3-Hydroxyanthranilic acid	neg	134.024892	C7H7NO3	Category 1
31	Linaroside	neg	475.125817	C23H24O11	Category 1
32	Lucidin 3-o-Primeveroside	neg	563.143963	C26H28O14	Category 1
34	Neochlorogenic Acid	neg	385.114309	C16H18O9	Category 1
35	Regaloside D	neg	399.130001	C18H24O10	Category 1
38	Mangiferin	neg	403.068546	C19H18O11	Category 1
39	4-Hydroxybenzoic Acid	neg	275.053901	C7H6O3	Category 1
41	Hesperetin	neg	301.072258	C16H14O6	Category 1
44	p-Hydroxy-5,6-dehydrokawain	neg	275.092743	C14H12O4	Category 1
45	Rugosinone	pos	354.096846	C19H15NO6	Category 1
49	DL-Menthol	neg	201.150038	C10H20O	Category 1
50	Teuclatriol	neg	301.202722	C15H28O3	Category 1
52	Monodictyxanthone	neg	251.035336	C15H10O5	Category 1
54	alpha-Zearalenol	neg	301.145332	C18H24O5	Category 1
55	trans-2-Undecenoic acid	neg	165.128749	C11H20O2	Category 1
57	Isosclerone	pos	196.096718	C10H10O3	Category 1
59	1-Deoxynojirimycin	pos	204.085524	C6H13NO4	Category 1
60	Beta-Zearalanol	neg	303.160851	C18H26O5	Category 1
63	Arecoline	pos	156.101649	C8H13NO2	Category 2

65	2,6-Diaminoheptanedioic acid	pos	232.128736	C7H14N2O4	Category 2
66	Tubercidin	neg	265.09315	C11H14N4O4	Category 2
67	Coriandrin	neg	321.062543	C13H10O4	Category 2
68	2-Hydroxypyridine	pos	113.070957	C5H5NO	Category 2
69	Gallic Acid	neg	169.014481	C7H6O5	Category 2
70	2-Tridecanone	neg	243.196961	C13H26O	Category 2
71	Medicagenic Acid	neg	501.323151	C30H46O6	Category 2
72	2-keto palmitic acid	neg	269.212564	C16H30O3	Category 2
75	beta-Hydroxymyristic acid	neg	243.196923	C14H28O3	Category 2
76	Cabralealactone	pos	431.315337	C27H42O4	Category 2
77	Cholesterol	pos	369.351102	C27H46O	Category 2
78	Taurolithocholic acid	pos	448.290141	C26H45NO5S	Category 2
81	Lecithin	pos	758.56855	C42H80NO8P	Category 2
83	1-Butanol	pos	74.0968215	C4H10O	Category 2
84	Methyl Gallate	neg	183.030293	C8H8O5	Category 2
86	Asterbatanoside A	neg	411.129947	C19H26O11	Category 2
87	Furfuryl acetate	pos	141.054422	C7H8O3	Category 2
89	Gentianine	neg	215.083002	C10H9NO2	Category 2
90	5-o-Feruloylquinic Acid	neg	367.103885	C17H20O9	Category 2
92	Hippuric acid	neg	178.051251	C9H9NO3	Category 2
97	Puerarin	pos	417.117644	C21H20O9	Category 2
98	Daidzin	neg	415.104935	C21H20O9	Category 2
101	4-Methyl-2-oxovaleric Acid	neg	189.077221	C6H10O3	Category 2
105	Ellagic Acid	neg	300.999718	C14H6O8	Category 2
106	Methyl L-pyroglutamate	neg	178.02746	C6H9NO3	Category 2
107	Nonane-4,6-dione	neg	155.108043	C9H16O2	Category 2
108	Demethylbellidifolin	neg	213.02313	C13H8O6	Category 2
109	Senkyunolide s	neg	221.082314	C12H16O5	Category 2
110	4-Allylpyrocatechol	neg	149.061002	C9H10O2	Category 2
113	Jaceosidin	neg	329.067528	C17H14O7	Category 2
115	zerumboneoxide	pos	427.259823	C15H22O2	Category 2
118	Senkyunolide G	neg	207.102923	C12H16O3	Category 2
120	Atractylon	pos	239.142622	C15H20O	Category 3
121	Ricinoleic acid	pos	281.247061	C18H34O3	Category 3
122	Indoxyl sulfate	neg	212.002594	C8H7NO4S	Category 3
124	Pseudoecgonine	pos	186.112114	C9H15NO3	Category 3
129	Caesalmin E	pos	534.273403	C26H36O9	Category 3
132	Kurarinol	pos	507.26214	C26H32O7	Category 3

137	Lancifodilactone C	neg	525.214998	C29H36O10	Category 3
